# Glucose Metabolism on Tumor Plasticity, Diagnosis, and Treatment

**DOI:** 10.3389/fonc.2020.00317

**Published:** 2020-03-06

**Authors:** Xiaoping Lin, Zizheng Xiao, Tao Chen, Steven H. Liang, Huiqin Guo

**Affiliations:** ^1^Department of Nuclear Medicine, Sun Yat-sen University Cancer Center, Guangzhou, China; ^2^State Key Laboratory of Oncology in South China, Collaborative Innovation Center for Cancer Medicine, Sun Yat-sen University Cancer Center, Guangzhou, China; ^3^Division of Nuclear Medicine and Molecular Imaging, Department of Radiology, Harvard Medical School, Massachusetts General Hospital, Boston, MA, United States; ^4^Department of Thoracic Surgery, Beijing Sijitan Hospital, Capital Medical University, Beijing, China

**Keywords:** metabolism, tumor, plasticity, glucose, Warburg effect, diagnosis, treatment

## Abstract

Malignant cells support tumor proliferation and progression by adopting to metabolic changes. Tumor cells altered metabolism by increasing glucose uptake and fermentation of glucose to lactate, even in the aerobic state and the presence of functioning mitochondria. Glucose metabolism in tumor plasticity has attracted great interests by clinicians and scientists in the past decades. This review discusses the previous and emerging researches on the tumor plasticity altered by changing glucose metabolism in different cancer cells, including cancer stem cells (CSCs). In addition, we summarize the rising applications of glucose metabolism in tumor diagnosis and treatment. Our objective is to direct future investigation on this altered metabolic phenotype and its application in patient care.

## Introduction

The characteristics of malignant cells, including sustaining cell proliferation, escaping cell death, attaining immortality by inducing new blood vessel formation and promoting tumor cell invasion and metastasis, were summarized in the year 2000 (1). After one decade of conceptual advance, two emerging traits were added—altering energy metabolism and evading immune demolition (2). In recent years, increasing number of studies focus on the alteration of energy metabolism, that allows tumor cells to survive and spread even in challenging conditions. However, a paradigm shift has occurred adding to our knowledge of the function of glycolysis in glucose metabolism over the last decade (3). In this review, we attempt to provide a better understanding of glucose metabolism in tumor plasticity which may contribute to the design and outcome of novel diagnostics and treatment strategies.

## Glucose Metabolism and the Warburg Effect in Tumors

Normally, glucose is processed by glycolysis to generate ATP and pyruvate. Then the ribose 5-phosphate and NADPH were produced through the pentose phosphate pathway (PPP), or enter into the tricarboxylic acid (TCA) cycle in mitochondrion. Glucose-derived citrate is converted to acetyl-CoA, oxaloacetate (OAA), or a-ketoglutarate (a-KG). Glutamine is deaminated to form glutamate, which is processed to produce a-KG for use in the TCA cycle.

The main pathway of glucose metabolism in cancer cells is aerobic glycolysis, termed Warburg effect (4). In cancer cells, glucose uptake and the production of lactate was dramatically increased, even in the presence of oxygen and fully functioning mitochondria (5). This classic type of metabolic change provides substrates required for cancer cell proliferation and division, which is involved in tumor growth, metastatic progression and long-term survival (5–8). It must be emphasized that both glycolysis and mitochondrial metabolism are crucial to cancer cells in the Warburg Effect (5).

Glucose metabolism in tumor is governed by both oncogenes and cancer-producing factors (6). Metabolic reprogramming of cancer cells is regulated by transcription factors that include c-*Myc, p53* and hypoxia-inducible factor (HIF) 1α (9). The reprogramming is a complex interaction of various signaling pathways, such as Notch, Akt, phosphoinositide-3-kinase (PI3K), PTEN, mammalian target of rapamycin (mTOR), and AMP-activated protein kinase (AMPK) (10, 11).

c-*Myc* can stimulate glycolysis, glutaminolysis, and nucleotide synthesis (12). c-*Myc* mediated glucose metabolic reprogramming primarily on mitochondrial aerobic metabolism (13). Glycolysis can be promoted by c-*Myc* through direct induction of glycolytic-associated enzymes (14). Besides, mitochondrial biogenesis can be promoted by c-*Myc* with stable function and the number of mitochondria in tumor cells.

*p53* is the main adverse regulator during tumor metabolic reprogramming (15). *p53* inhibits glycolysis by inducing glycolysis and apoptosis regulator (TIGAR), inhibiting phosphoglycerate mutase (PGM) to upregulate expression of TP53, and repressing glucose transporter (GLUT)-1 and GLUT -4 (6, 16–18). Also, *p53* can alter oxygen consumption and the synthesis of cytochrome c oxidase 2 (SCO2) protein, which is critical for regulating the cytochrome c oxidase(COX) complex (19). Moreover, *p53* promotes mitochondrial glutaminase (GLS2) and limits glutaminolysis in response to oxidative stress or DNA damage (20).

HIF-1 is a heterodimeric protein that could alter various genes coded for enzymes involved in glucose metabolism. The phosphatidylinositol 3-kinase (PI3K) and ERK mitogen-activated protein kinase (MAPK) pathways affect HIF-1α protein synthesis. In glucose metabolism, glyceraldehyde-3-P-dehydrogenase (GAPDH), GLUT-1, hexokinase (including HK1 and HK2), autocrine motility factor/ (AMF/GPI), enolase 1(ENO1), plasminogen activator receptor (TPI), Pyruvate kinase(PKM), 6-phosphofructo-2-kinase/fructose-2,6-biphosphatase-3(PFKBF3, PFKL, PGK1), and LDHA can be transcriptionally activated by HIF-1 (21).

## The Impact of Glucose Metabolism on Tumor Plasticity

Tumor cells need to survive drastic changes in the microenvironment such as hypoxia, nutrient storage, and acidic pH (22). A huge number of cancer cells show remarkable plasticity in metabolic adaptation. The reprogrammed glucose metabolism allows cancer cells to satisfy high proliferation requests. In addition, it provides some survival and growth advantages, including high carbon source for anabolism, rapid ATP availability to supply the energy, abundant lactic acid to increase the redox status (NADPH) via the glycine–serine pathway (6–8). Lactic acid induces metabolic “dormancy” and is involved in EMT and tumor immune response by reducing pH in the tumor environment (5, 8, 23–25). To manage all the situations above, cancer cells must maintain a balance to deliver adequate energy with constrained resources and to meet the biosynthetic demands of proliferation. Though oxidative phosphorylation(OXPHOS) would be the best energy provider, the physiological reality is that both OXPHOS and glycolysis collaborate to produce ATP under the local oxygen concentration. Coordinate results are net increments in glucose utilization and lactic acid secretions. This process is known as the glycolytic switch, which is corresponding to uncoupling glycolysis from OXPHOS (26).

### Glucose Metabolism and Cancer Cell Proliferation

Cell proliferation requires expanded uptake of supplements, lifted flux through biosynthetic pathways, support of metabolic intermediates, and proceeded recovery of cofactors required to supply energy or reducing equivalents for reactions. Cancer cells preferred aerobic glycolysis for cell proliferation. In addition, aerobic glycolysis produces metabolic precursors that are essential for rapid cell proliferation (25). As proliferation is the key feature of cancer cells, aerobic glycolysis allows cancer cells to meet the requirements of generating enough ATP and biosynthetic precursors. The goal of aerobic glycolysis is to preserve high levels of glycolytic intermediates to maintain anabolic reactions in cells instead of generating lactate and ATP. Thus, it may explain why increased glucose metabolism happens in proliferating cancer cells (26).

The biosynthesis in proliferating cells requires building blocks for the synthesis of nucleotides, lipids, and non-essential amino acids—those that glycolytic intermediates can supply (27). The PPP can produce the reducing equivalents in the form of NADPH molecules and generates nucleotide and lipid precursors. The TCA cycle can generate acetyl-CoA and glutamine and drive them into the cytosol. As a result, the anabolic metabolism of amino acids and lipids is supplied by both glycolysis and the TCA cycle within mitochondria (27). NAD+ is an essential cofactor of nucleotide and amino acid biosynthesis. The maintenance of biosynthesis in proliferating cells demands the regeneration of NAD+. The conversion of pyruvate to lactate can partially produce NAD+ (28). Because cells use as much as 10% of their entire proteome and half of all of their metabolic genes to produce proteins involved in glycolysis, the cost of using Warburg Effect in aerobic glycolysis as a tradeoff to promote biosynthesis is vast (29). Mitochondrial functions occur concomitantly with the aerobic glycolysis and limiting mitochondrial activity may not occur during the Warburg Effect (5). Under energy stress conditions, the apparent shift from glycolysis to OXPHOS by mitochondrial elongation contribute to tumor survival. Remodeling of mitochondrial morphology is a remarkable protection of tumor cells from stress (30).

### Glucose Metabolism and EMT

EMT is a process that involves a high level of cellular plasticity. EMT is often activated during cancer cell invasion, systemic dissemination, and metastasis (31, 32). EMT is an important step preceding to invasion and metastasis in tumor cells. Epithelial cells lose the junctions among cells and their polarized organization during EMT. They change cytoskeletal organization, transform the shape, and acquire mesenchymal characteristics, such as fibroblast-like cell morphology and increased capability of invasion and migration (32, 33).

The plasticity of glucose metabolism is important in EMT (34). The genes and biochemical mechanisms impact glucose metabolism during EMT of cancer cells. Crosstalk network has been explored between EMT and cancer metabolism (35). PI3K-AKT-mTOR, EGFR-RAS-MAPKs, and JAK2-STAT3 signaling pathways can mediate EMT (36). HIF1α, Myc and FOXM1 regulate both metabolism and EMT (37). LKB1 / AMPK (Liver kinase B1/AMP-activated protein kinase) downregulates SNAIL and ZEB1and inhibits the invasion and migration of tumor cells, by regulating FOXO3, TGF- β, NF-κB, AKT, and mTOR signaling pathways (34).

Glucose transporters, especially GLUT-1 and GLUT-3, promote tumor progression by increasing glucose influx and activating downstream molecular pathways (38). GLUT-1 increases matrix metalloproteinase 2 (MMP-2) *in vitro* and *in vivo*, which contributes to EMT and cellular invasiveness (34, 39). GLUT-3 gene could be activated by ZEB1, which is an EMT marker (34). HK2 is a well-known hypoxia-inducible gene that can induce EMT (34). PFK increases glycolytic flux and EMT by maintaining this glycolytic phenotype in cancer cells *in vitro*. Pyruvate kinase M2 (PKM2) can prompt EMT both by metabolic and non-metabolic mechanisms (40). PKM2 increases glucose uptake and lactate production to support cell survival and invasion (41).

Besides the enzymes involved glucose metabolism, altered mitochondrial function also contributes to EMT induction (34). Tumor cell migration and metastasis was stimulated by abnormal TCA cycling coupled with mitochondrial superoxide production (42).

### Glucose Metabolism and Cancer Stem Cells

Cancer consists of mainly stem cells (CSCs) and non-CSCs (2). CSCs have the potentials of self-renew and tumor initiation (43, 44). CSCs adapt to metabolic plasticity, which is determined by the factors present in the tumor microenvironment (TME). Metabolic plasticity allows these cancer stem cells to switch between OXPHOS and glycolysis (45). Only complete oxidation through the TCA cycle cannot supply enough anabolic precursors such as pyruvate and glutamine. CSCs prefer to rely on glycolysis and the PPP and devolve mitochondrial infrastructure and function. This predominantly glycolytic metabolism offers sufficient energy to support the basic needs of CSCs. The maturation of metabolic network matches the increasing energy demands of specialized progeny cells. The oxidative metabolism infrastructure contains mitochondrial biogenesis and maturation, and networks of the TCA cycle and electron transport chain. A concurrent rise in mitochondrial ROS may prime CSCs for lineage differentiation (46). The main difference between cancer cells and CSCs is the metabolic shift and mitochondrial resetting. CSCs display the metabolic change and mitochondrial resetting into precise bioenergetic states and lose the unique metabolic phenotypes after differentiation. Compared with differentiated neoplastic cells, CSCs exhibit a more prominent Warburg effect. Aerobic glycolysis may produce enough glycolytic intermediates into the PPP to supply molecules that are necessary for anabolic metabolism and growth of CSCs (47). In CSCs, the oxidative metabolism and mitochondrial structure are altered to commit glycolysis (48). Aerobic glycolysis is one of the important aspects in maintaining CSCs and inducing their differentiation. Specifically, aerobic glycolysis is critical in preserving the stemness of CSCs, while switching to oxidative metabolism is the characteristic of stem cell differentiation. Besides, aerobic glycolysis is essential to the properties of CSCs (46). Multiple regulatory factors including metabolic enzymes promoted the metabolic plasticity of stem cells in breast cancers. The metabolism-regulating genes and epigenetic factors that regulate glucose metabolism might also regulate the expression of EMT (49). An enhanced Warburg effect was observed in metastatic prostate cancers (44).

Moreover, metabolic flexibility diverge the fates CSCs, which include dormancy to minimize stress damage, proliferation and self-renewal to preserve progenitor pools, and pedigree specification for tissue regeneration (48). Depending on the metabolic characteristics of the tumor cells of origin, isogenic glioma stem cells (GSCs) exhibits heterogeneity in metabolic characteristics. They can be divided into mitochondrial and glycolytic phenotypes. Cells of the mitochondrial type consume more oxygen and maintain a higher ATP content; those of the glycolytic type consume more glucose and produce more lactate. Both metabolic phenotypes are independent and stable. They can coexist within a given tumor. The environmental factors further influence the metabolic preferences of these cells. For example, CSCs that rely on OXPHOS can switch to aerobic glycolysis in response to metabolic stress (50).

### Glucose Metabolism and Tumor Microenvironment (TME)

In addition to the inherent alterations in the tumor cells, the metabolic competition and cooperation among the TME components support tumor proliferation, progression, and therapeutic resistance (9). TME consist of different types of cells, that include cancer associated fibroblasts (CAFs), non-cancer cell stroma, immune cells, and endothelial cells (51). The TME forms a pro-tumorigenic cocoon around the tumor cells. Intrinsic traits (e.g., genetic programs in cancer cells) and extrinsic factors (e.g., nutrient availability, oxygen tension, pH) contribute to the deregulated metabolism in TME. TME enforces metabolic plasticity to adapt to hypoxic and acidic environment, nutrient deprivation and competition, oxidative stress, and immune surveillance (52). Reprogramming of the metabolism occurs in tumor and non-tumor cells. The interactions and competitions in TME components guarantee the steady supply of nutrients and molecules for tumor growth even under hypoxic conditions. Metabolic reprogramming also affects TME. Hypoxia inhibits this process by upregulating PDK1 and LDHA (53, 54). When HIF1α is activated in CAFs, the activity of mitochondria drops and lactate production increases. This is consistent with a glycolytic phenotype. It leads to slow down metabolism in the microenvironment (55). Lactate accumulation resulted from continuous activation of glycolytic and LDHA enzymes leads to a low-pH microenvironment during tumor progression. Nutrient deficiency is another microenvironment stress that cancer cell often encounters. Numerous studies have shown that reprogramming of glucose metabolism upon nutrient starvation in tumor cells occurs in order to use energy to support their growth (56–61).

The reverse Warburg Effect was proposed in the last decade (62). The energy-rich metabolites of aerobic glycolysis, such as lactate and pyruvate, are generated by cancer associated fibroblasts and taken up and used in the TCA cycle in mitochondria of epithelial cancer cells. Thus, efficient energy production such as ATP generation, which leads to increase cell proliferation and reduce cell death (62). The reverse Warburg effect can benefit tumor cells by cooperative utilization of oxygen between stromal cells and tumor cells (55). Cancer cells induce stromal cells to undergo “aerobic glycolysis.” Their products are returned to the cancer cells to be used for mitochondrial OXPHOS (63). The metabolic heterogeneity in TME allows cancer and stromal cells to exchange metabolites between them to maintain maximal cellular growth (18).

The immune system plays an important role in TME. The immune cells in the TME can detect and eliminate the abnormal cells or tumor cells and protect the body from damage caused by tumor cells (64). Tumor cells activate immune cells, and the activated innate or adaptive immune cells can maintain homeostasis (9, 65). The immune system affects cancer survival and progression (23, 66). Tumor cells develop different mechanisms to escape the immune response. They include strategies at the genetic, epigenetic and metabolic levels. It implements to resist immune recognition and decrease apoptosis (67).

There is a complex interaction between malignant cells and immune cells in the tumor stroma (66). Metabolism regulates tumor cells to escape immune surveillance and to coexist with stroma cells. Tumor and other different types of cells in the TME compete for nutrient. Inflammation induced by oncogene in the tumors promotes adaptive metabolic changes in the surrounding non-tumor cells to secrete metabolites. Tumors use these metabolites as alternative nutrient sources to meet their increasing demands for anabolic function (68).

Metabolism is essential to lymphocyte for its development, function and inducing tolerance (69). T cells prefer glycolysis upon activation, though they had lower glycolytic flux when resting. Activated by TCR- and CD28-mediated co-stimulation, T cells switch to a rapid increase in glucose uptake and glycolysis (69–71), as well as glutaminolysis (69, 72, 73). In contrast, the activation of T cells and dendritic cells can be inhabited by lactate accumulation (74, 75). Lactate prevents cytokine release and monocyte migration, while promotes the formation of tumor-associated macrophage 2 (TAM2) phenotype. It leads to upregulate the expression of arginase 1, promote immune escape and tumor progression (25, 76–78). Lactate reduces the production of IFN-γ from T cells and NK cells, and decreases the level of nuclear factor of activated T cells (NFAT), which also contributes to immune escape and tumor progression (79, 80). As antigen presenting cells and a source of cytokines, B lymphocytes also have a critical role in antitumor immunity (81, 82). B cells upon activation use both glycolysis and OXPHOS, which is different from T cells. However, the deletion of GLUT-1 or inhibition of glycolysis in B cells suppresses antibody production *in vivo* (69, 70).

## Clinical Application of Targeting Glucose Metabolism in Tumor

Attempts to target the glucose metabolism, especially on Warburg effect, for cancer diagnosis and therapy emerges in the past decades and is still in developing.

### Application of Glucose Metabolism in Cancer Diagnosis

Many different types of human tumors dramatically enhanced uptake and use of glucose. Since 1976, positron emission tomography (PET) with a radiolabeled analog of glucose (^18^F-fluorodeoxyglucose, FDG) was applied to non-invasively visualize glucose uptake in human body. This tracer is the most impressively clinical utility of the Warburg effect (2, 6). Increased FDG implies high glucose uptake which is an indication of the glycolytic switch. It provides information about the pathologic differentiation and precise stageing of tumors, predicts treatment response and gives an indication of overall prognosis (83). The positron-emitting radionuclide fluorine-18 replaces the normal hydroxyl group at the C-2 position in the glucose molecule. After injection into the body, the tracer is transported into cells by glucose transporters, especially GLUT-1 and GLUT-3, followed by phosphorylation with the hexokinase, especially HK2, to produce ^18^F-FDG-6-phosphate (^18^F-FDG-6-p). ^18^F-FDG-6-p cannot be released from the cell and is trapped in the cytoplasm. Because of the lack of the 2'hydroxyl group, ^18^F-FDG-6-p cannot further proceed to glycolytic pathway (84). PET scanner detects the radioactive decay of ^18^F-FDG-6-p and form the body images of the distribution of ^18^F-FDG. Thus, the presence of living malignance can be identified by the accumulated amounts of ^18^F-FDG-6-p (84, 85). The sites and the semi-quantitative analysis of high glucose uptake (e.g., standard uptake value, SUV) in the whole body can be identified. In the vast majority of malignance, glucose is trapped in cancer cells more than normal tissues with the exception of the brain and brown fat. It relates to the metabolic characteristics at the tumor site (42). Owing to the limitation of spatial resolution and some particular pathology subtypes(e.g., signet ring cell carcinoma, well-differentiated cancer), the sensitivity and specificity varied across different applications using^18^F-FDG–PET (26, 85).

PET imaging tracers are able to detect the PKM2, such as N,N-diarylsulfonamide (DASA) compounds bind to PKM2. ^11^C-labeled analog of DASA-23 was applied to the orthotopic U87 and GBM39 patient-derived tumors in preclinical models of glioblastoma multiforme and to monitor the response of the PKM2 activator TEPP-46 in GBM39 tumors (86).

An analog of glutamine, 4-^18^F-(2S,4R)-fluoroglutamine (^18^F-FGln), is taken up by cancer cells *in vitro* and its specific uptake can be detected on PET imaging in mouse xenograft model *in vivo*. Thus, the glutamine metabolism in gliomas and its uptake can be evaluated (87). Other investigational PET agent, such as 5-^11^C-(2S)-glutamine (87, 88), was also exploited for its ability to take up and retain glutamine in some tumors (87).

As another device in molecular imaging, a few agents have been developed to detect the glucose metabolism in magnetic resonance (MR). The hyperpolarized agents can form the better imaging for remarkable enhancement compared to conventional MR imaging. Magnetic resonance spectroscopy(MRS) can image the conversion of ^13^C-labeled pyruvate to lactate in patients (83). It is possible to identify malignance and monitor treatment response by evaluating the distribution of ^13^C-pyruvate and the altered ^13^C -lactate/^13^C -pyruvate in preclinical MRS study in prostate cancer and glioma (83, 89). Some other targetable processes in glucose metabolism, such as the detection of 2HG, can also be used for new imaging technology (90, 91).

### Application of Glucose Metabolism in Cancer Treatment

To date, various agents involved in glucose metabolism are actively investigated as novel targets with therapeutic potential (Table 1). It helps to overcome drug resistance or increase the efficacy of current combination therapy (88, 109). Compared to diagnosis, targeting glucose metabolism in treatment seems faint due to their efficacy or safety concern. Several drugs with efficacy confirmed and multi-targets, as well as some old non-chemotherapeutic drugs with new aspects of inhabiting tumor glucose metabolism will be discussed below.

**Table 1:** Metabolic modulators arising from the metabolic theory of cancer.

**Target**	**Therapy**	**Reference**
Glucose transporters (GLUTs)	2-deoxyglucose (2DG)	(92, 93)
	Phloretin	(49, 71)
	Silybin	(26, 94)
	Glutor	(95)
	STF-31	(94, 96)
	WZB117	(94, 96)
	Fasentin	(97, 98)
Hexokinase (HK)	2-deoxyglucose (2DG)	(92, 93)
	3- bromopyruvate (3BP)	(26, 96)
	Lonidamine(LND)	(99, 100)
	Methyl jasmonate	(94, 97)
Glyceraldehyde 3-phosphate dehydrogenase(GAPDH)	3- bromopyruvate (3BP)	(26, 96)
	Ornidazole, a-chlorohydrin	(24)
Phosphofructokinase (PFK)	3-(3-pyridinyl)-1-(4-pyridinyl)- 2-propen-1-one (3PO)	(26, 93)
Pyruvate kinase-M2 (PK-M2)	TLN-232/CAP-23	(26, 92, 94)
	Shikonin	(24, 26)
	Alkannin	(24, 26)
	TEPP-46	(24, 86)
	DASA-58	(24, 101)
	ML-265	(17, 102)
	Oleanolic acid (OA)	(103)
	dimethylaminomicheliolide(DMAMCL)	(104)
Lactate dehydrogenase (LDH)	2,3-dihydroxynaphtalen-1-carboxylic acid (FX11)	(24, 94)
	N-hydroxy-2-carboxy-substituted indoles (NHI)	(24, 105)
	Oxamate	(24)
	R-(-)-gossypol / AT101	(24)
	3-hydroxyisoxazole-4-carboxylic acid (HICA)	(26, 34)
	Gossypol/Galloflavin	(52)
	5 designed peptides(QLYNL, LIYNLL, IYNLLK,KVVYNVA, and KVVYNV)	(52)
	1-(Phenylseleno)-4-(Trifluoromethyl)	(52)
	Benzene(PSTMB)	(24, 105)
	Oxamate(siRNA LDHA gene, Galloflavin)	(92, 96)
	FK866	(26, 52)
	AZD3965	(24, 52)
	AR-C155858	(95, 106)
	a-cyano-4-hydroxycinnamic acid (CHC), 4,4-diisothiocyanatostilbene-2,2′ -disulphonic acid(DIDS), Quercetin	(52, 106)
	BAY-8002	(52)
	Syrosingopine	(52)
Isocitrate dehydrogenase (IDH)	AGI-5198	(102, 107)
	AGI-6780	(102, 108)
PDK	TKIS	(52, 102)
	Dichloroacetate (DCA)	(14, 49)

GLUTs control the influx of glucose, especially GLUT-1. Several GLUT-1 inhibitory agents, including WZB117 and STF-31 have been tested (94, 105). STF-31 was effective in decreasing glucose uptake, inducing cell apoptosis and inhibiting tumor progression. However, STF-31 has a narrow therapeutic potential due to its molecular restriction (110, 111). WZB117 can effectively inhibit glucose uptake, cell proliferation, and tumor progression both *in vivo* and *in vitro*. However, its efficacy remains in doubt (105). A glucose-conjugated LDH inhibitor, glucose-conjugated methyl ester (NHI-Glc-2), is a promising compound. It is a weaker inhibitor than the N-OH methyl ester (NHI-2) on the isolated enzyme. It can increase the glucose uptake by exploiting the GLUT-1 overexpression, reduce lactate production and decrease proliferation of cancer cells (105).

Currently, inhibition of lactate transport is being tested as an alternative approach. But LDHA inhibitors had some limitations, such as high toxicity, low drug exposure or a lack of LDHA dependence in human tumor inhibitors (112). Nevertheless, an inhibitor of human LDH isoforms, Galloflavin is a natural phenol derivative and a product of gallic acid oxidation (105, 113). The analogs of Gossypol, a natural component extracted from the cotton seeds has been screened for small molecular inhibitors specific for LDHA. 3-dihydroxy-6-methyl-7-(phenylmethyl)-4-propylnaphthalene-1-carboxylic acid (FX11) was proved to effectively inhibit proliferation of cancer cells *in vitro* and *in vivo* by enhancing the levels of oxidative stress. Another isoform-specific inhibitors of LDHA, N-hydroxyindole-based compounds, can compete with its substrate pyruvate and the cofactor, NADH (92, 97).

The reversal reprogramming in IDH mutant tumors seems more successful (114, 115). The mechanism of 2HG-mediated transformation may vary in different kinds of tumors. It may inhibit the dual metabolic flux of glycolysis and oxidative PPP (88, 116). In preclinical studies, the inhibition of mutant IDH has been shown to dramatically decrease the generation of 2HG and cause cancer cells to differentiate into normal cells (107, 108). An inhibitor of mutant IDH2, AG-221, has been put forward in early phase clinical trials (113).

New compounds also target dual inhibition, such as the inhibition of metabolic plasticity and metabolic rescue in cancer cells. Compounds targeting glucose, glutamine and lactate metabolism have been found to exert anticancer effects by inhibiting growth of tumor-associated endothelial cells (24). A new glucose uptake inhibitor, Glutor, targets GLUTs (GLUT-1, -2, and -3), diminishes glycolytic flux and selectively suppresses growth of a variety of cancer cells. Glutor combined with glutaminase inhibitor CB-839 synergistically inhibits the proliferation of colon cancer cells (95).

Compared to conventional cytotoxic therapy, modulation of particular targets with altered glycolytic metabolism would reduce treatment toxicity. A number of studies show that treatment combined with vitamin C leads to interfering with glycolysis and the TCA cycle and inhibits ATP and NADPH production. It can kill cancer cells by increasing oxidative stress and further inhibition in cancer cell survival and invasion. (117–122). Preclinical studies have shown that vitamin C at the concentration below 5 mM could prevent proliferation of cancer cells (123–125). Because of the anti-oxidant capacity, vitamin C can prevent the growth of circulating tumor cells (CTCs). Vitamin C also reduces pyruvate and glutathione (GSH). Extracellular matrix remodeling and cancer cell motility were reduced by vitamin C by boosting ROS levels. It can increase expression of E-cadherin, decrease expression of Snail and inhibit matrix metalloproteinases (MMPs) (105, 126, 127).

Metformin is a common drug for diabetes. The metformin/hypoglycemia combination has synergistic anti-neoplastic effects to decrease the pro-survival protein MCL-1 and cause cell death (128). Metformin combined with ritonavir targets OXPHOS, in particular, GLUT-4. It can effectively inhibit the AKT and mTORC1 phosphorylation and pro-survival mitochondrial complex I (MCL1) (116, 129).

Aspirin is a common pain reliever and anti-inflammatory drug. It also inhibits MCL1 activity (130). In the past two decades, its anti-neoplastic action has been investigated against different malignancies and tumor cell lines. Dalton's lymphoma (T-cell lymphoma) cells obtained from tumor-bearing mice treated by aspirin showed a change of expression of pH regulators MCT-1 and V-ATPase, as well as change in cell survival regulatory molecules including GLUT-1 (131). Aspirin can also modulate glucose uptake by depressing GLUT-1 through targeting NF-κB or NF κB/HIF1α signaling to inhibit proliferation (132).

## Conclusion

Despite the extensive study on cancer metabolism with interesting results accumulated in the last decades, questions are still arising. The key process of balance among glycolysis, TCA and other pathways of the glucose metabolism in tumor remains unclear, as it is the essential mechanism of Warburg effect. In addition, it should be considered to develop more tumor-specific tracers and drugs based on the metabolic switch in tumor cells, cancer stem cells or the interaction with immune system. The ideal drugs should only applied in tumor by blocking specific pathway(s) for its metabolic plasticity but not in normal tissues. Despite the emerging of metabolic enzymes or transporters inhibitors, the efficiency of targeting tumor glucose metabolism is being challenged. To explore the metabolic plasticity in cancer under intrinsic and extrinsic influences, tumorous glucose metabolism should be addressed. Nevertheless, with technological advances, it is expected that we will uncover many other unknown aspects of glucose metabolism in cancer and use them to benefit patient care.

## Author Contributions

XL and HG conceived and designed the study. XL wrote the first draft. ZX, TC, and SL wrote some sections of the article. HG edited the article. All authors read and approved the final version of submission.

### Conflict of Interest

The authors declare that the research was conducted in the absence of any commercial or financial relationships that could be construed as a potential conflict of interest.
